# Exposure to polystyrene nanoplastics impairs sperm metabolism and pre-implantation embryo development in mice

**DOI:** 10.3389/fcell.2025.1562331

**Published:** 2025-02-28

**Authors:** Yingdong Liu, Fengdan Hao, Haixin Liang, Wenqiang Liu, Yi Guo

**Affiliations:** ^1^ Shanghai Key Laboratory of Maternal Fetal Medicine, Shanghai Key Laboratory of Signaling and Disease Research, Centre for Assisted Reproduction of Shanghai First Maternity and Infant Hospital, Clinical and Translational Research Center of Shanghai First Maternity and Infant Hospital, Shanghai Institute of Maternal-Fetal Medicine and Gynecologic Oncology, Frontier Science Center for Stem Cell Research, School of Life Sciences and Technology, Tongji University, Shanghai, China; ^2^ Department of Pediatrics, Tongji Hospital, School of Medicine, Tongji University, Shanghai, China

**Keywords:** polystyrene, immune-inflammatory response, sperm motility, metabolic disruptions, DNA repair, embryonic development

## Abstract

**Introduction:**

Microplastics and nanoplastics are prevalent environmental contaminants. Recent reports indicate that polystyrene nanoparticles may adversely impact male reproductive health. This study aims to examine the effects of polystyrene exposure on sperm metabolism and the development of pre-implantation embryos.

**Methods:**

In this study, male C57BL/6 mice were orally gavage-administered polystyrene nanoplastics (60 nm, 20 mg/kg/day) for 35 days to assess their impact on male reproduction and early embryonic development. Experiments included testicular transcriptome analysis, sperm metabolomics, sperm motility and fertilization assays, embryonic ROS detection, and RNA sequencing of 2-cell embryos, revealing the adverse effects of polystyrene exposure on sperm metabolism and embryo development.

**Results:**

The results revealed that oral gavage of polystyrene to male mice induced a pronounced immune-inflammatory response in testicular tissue, reduced sperm motility, and significantly lowered the fertilization rate. Notably, sperm from treated mice exhibited substantial metabolic disruptions, affecting key pathways, including glycerophospholipid biosynthesis and DNA repair. After fertilization, embryos at the 2-cell stage suffered damage in apoptotic and DNA repair pathways, subsequently impairing early embryo development.

**Discussion:**

In conclusion, this study demonstrated that the oral gavage administration of polystyrene nanoplastics to male mice significantly affects male reproductive function, resulting in abnormalities in early embryonic development and alterations in associated gene expression profiles. These findings offer essential scientific insights for future research into sperm-mediated transgenerational effects and their impact on early embryonic development.

## 1 Introduction

Microplastics (MP) and nanoplastics (NP) are pervasive and unavoidable in the environment (He and Yin, 2024). Microplastics are considered to be tiny plastic fragments with sizes ranging from 1 μm to 5 mm (Frias and Nash, 2019). In addition, microplastics can also degrade into smaller nanoscale particles with sizes ranging from 1–1,000 nm (Gagne, 2019). Among these, polystyrene microplastics and nanoparticles (PS-MNP) contribute significantly to environmental pollution (Camerano Spelta Rapini et al., 2024). Polystyrene (PS) is particularly prevalent in everyday products such as packaging materials, insulation, and consumer goods, presenting potential health risks (Geyer et al., 2017). The impact of environmental pollutants on reproductive health has become a growing concern (Dusza et al., 2023; Hu et al., 2024). Research on polystyrene’s effects on mammalian reproductive health has predominantly focused on gametogenesis, including oogenesis and spermatogenesis (Camerano Spelta Rapini et al., 2024; Tanveer et al., 2023).

Maternal exposure to polystyrene through drinking water during pregnancy can adversely affect both the placenta and fetus, leading to morphological abnormalities in skeletal muscle tissue. Moreover, exposure to high concentrations (10 mg/L) of polystyrene results in significant reductions in both absolute and relative fetal weight (Chen et al., 2023). Cross-generational toxicity studies have shown that maternal exposure to microplastics can cause premature death in rodent offspring. Survivors exhibit a range of disorders, including metabolic dysfunction, reproductive impairment, immune system abnormalities, and disruptions in neurodevelopment and cognition (He and Yin, 2024).

Spermatogenesis occurs within the seminiferous tubules of the testes, the seminiferous epithelium is composed of germ and sertoli cells. Spermatogonia differentiate into spermatocytes through mitosis, which then undergo meiosis to form round spermatids, ultimately maturing into sperm. This intricate process is regulated by endocrine, paracrine, and autocrine signals, and is influenced by various environmental and lifestyle factors (Gao et al., 2024; Makela et al., 2019; Saitou and Hayashi, 2021). Research has shown that male mice were oral gavage-administered polystyrene nanoparticles (25 nm, 50 nm, and 100 nm) for 56 days. Exposure to all three nanoparticle sizes resulted in reduced male fertility and potential infertility. These nanoparticles accumulated in the testes, inducing oxidative stress, altering the expression of genes associated with apoptosis and inflammation, and disrupting energy metabolism (Xu et al., 2023). Additionally, exposure to polystyrene microplastics led to developmental abnormalities in testicular tissue and decreased sperm count and motility in adult male mice. These effects may be attributed to the disruption of the Hippo pathway and abnormal cytokine secretion induced by polystyrene microplastics in the testes (Zhao et al., 2023). However, the impact of polystyrene exposure on sperm metabolite levels and its effects on early embryonic development and gene expression remain unclear.

In this study, male mice were exposed to polystyrene via oral gavage, resulting in an increased immune-inflammatory response in testicular tissue, alongside significant DNA damage in the testes. Notably, marked changes were observed in the metabolic profile of sperm from exposed mice, including alterations in metabolite levels related to glycerophospholipid biosynthesis and DNA damage repair. Furthermore, significant DNA damage was detected in sperm-derived early embryos, accompanied by disruptions in apoptosis and lipid metabolism at the 2-cell embryo stage. Overall, our findings suggest that polystyrene exposure in male mice leads to dysregulation of testicular gene expression and sperm metabolism, which in turn impacts lipid metabolism and oxidative stress pathways in early embryos, thereby reducing early embryo development. This study offers both theoretical and practical insights for future research into the mechanisms and biological effects of polystyrene.

## 2 Materials and methods

### 2.1 Polystyrene oral gavage treatment in animals

In this study, 10- to 12-week-old male C57BL/6 mice were divided into two groups (n = 10 each group). The experimental group received daily oral gavage of polystyrene green fluorescent microspheres (60 nm, 20 mg/kg/day) for 35 days. The control group received an equal volume of ultrapure water (ddH_2_O). All experimental procedures and animal breeding protocols were conducted in accordance with the ethical guidelines for the use of experimental animals at Tongji University (TJBG14924101).

### 2.2 Collection of oocytes

To perform superovulation in female mice, inject 5 IU of PMSG (San-Sheng, China) intraperitoneally into 8- to 10-week-old C57BL/6 female mice, followed by an injection of 6 IU of HCG (San-Sheng) 48 h later. Cumulus-oocyte complexes were collected from the oviducts 13–14 h after hCG injection. Next, place the cumulus-oocyte complexes in G-IVF to prepare for fertilization.

### 2.3 Sperm capacitation and *in vitro* fertilization

Male mice from both groups were euthanized by cervical dislocation. After euthanasia, the mice were placed in a dorsal position, and the epididymides were carefully removed using eye forceps. The epididymides and part of the vas deferens were dissected with scissors and placed in a dish. Using ophthalmic forceps, sperm were gently squeezed from the vas deferens, starting from the base and moving toward the head of the epididymis. An injection needle was used to puncture the epididymal head and extract the sperm. The sperm were then incubated in a G-IVF medium (Vitrolife, Sweden) for 20–30 min in a culture incubator.

After incubation, a small volume of sperm was collected from the upper layer with a pipette and transferred into an IVF culture dish containing cumulus-oocyte complexes. Once fertilization was complete, any remaining sperm were washed off the fertilized oocytes, and the fertilized egg were transferred into the G1-PLUS medium (Vitrolife, 10128) for further culture.

### 2.4 Testing of sperm motility

Remove the sperm from the tail of the mouse epididymis (Section 2.3). Place the sperm to be tested in a 35°C water bath for 10–15 min. Take 10 μL of sperm and transfer it to preheated CASA special glass slides and cover slides. Drop the sperm onto the glass slide, cover them with the cover slide, and let them stand for about 5 s for observation (n = 5 each group). Use the microscope equipped with CASA to locate sperm and observe them in real-time using computer.

### 2.5 Detection of sperm and embryonic reactive oxygen species

To assess oxidative stress of sperm, DCFH-DA (Beyotime, S0033S) was diluted in the DPBS at a 1:1000 ratio, resulting in a final concentration of 10 µM. Incubate the upstream sperm in the solution for 20 min at 37°C. After incubation, the sperm were washed three times with DPBS and examined under a microscope (n = 3 each group).

Similarly, DCFH-DA was diluted in the G1-PLUS medium at a 1:1,000 ratio, resulting in a final concentration of 10 µM. The embryos were incubated in this solution for 20 min at 37°C to allow sufficient interaction between the probe and the embryos. After incubation, the embryos were washed three times with fresh culture medium droplets and examined under a microscope. Fluorescence signals from each embryo were quantified using ImageJ software, and statistical analysis was performed using an unpaired t-test. A *p*-value of < 0.0001 was considered statistically significant (^∗∗∗∗^ *p* < 0.0001).

### 2.6 TUNEL assay

Fix 4-cell embryos with 4% paraformaldehyde at room temperature for 1 h. Wash once with 0.5% BSA-PBS. Next, the embryos were permeabilized with 0.5% TritonX-100 and incubated at room temperature for 20 min. Prepare TUNEL staining solution using TdT enzyme (5 μL) and fluorescent labeling solution (45 μL). Wash twice with 0.5% BSA-PBS. Add 50 μL TUNEL detection solution and incubate at 37°C in the dark for 60 min. Wash twice with 0.5% BSA-PBS. Detect the fluorescence intensity of embryos under a fluorescence microscope.

### 2.7 Detection of untargeted metabolomics

Sperm acquisition involved sperm capacitation and *in vitro* fertilization (IVF). The upstream sperm were then washed 2 to 3 times with 0.5% BSA-PBS at 1,500 rpm for 5 min. Following this, the sperm pellet was dissolved in a 1:1 solution of acetonitrile and water, analyzed, and separated using ultra-high-performance liquid chromatography (UPLC) with an ACQUITY UPLC BEH amide column. To ensure data reliability, quality control (QC) samples were introduced during the analysis. Further sample analysis was performed using quadrupole time-of-flight mass spectrometry (QToFMS; Triple TOF 6600; AB Sciex GmbH) in both positive and negative ion modes of electrospray ionization (ESI). Metabolites were identified by comparing their mass-to-charge ratio (m/z) accuracy (within 25 ppm) and MS/MS spectra to an internal database of established standards. Orthogonal Partial Least Squares Discriminant Analysis (OPLS-DA) was performed using the R software package (Wang et al., 2024). Differential metabolites were identified based on a predictive variable importance (VIP) threshold >1, and the results were validated with an adjusted P-value <0.05. Metabolome enrichment analysis was carried out using MetaboAnalyst 6.0 software (Pang et al., 2024). The detection of untargeted metabolomics was performed by Applied Protein Technology Co. (Shanghai, China).

### 2.8 Extraction of RNA from testicular tissue

Testicular tissue cells were thoroughly dissolved in a Trizol solution. To this, 200 μL CHCl_3_ was added, followed by vortexing and standing for 5 min. The mixture was then centrifuged at 13,000 rpm for 15 min at room temperature. The supernatant was collected, and an equal volume of isopropanol was added. The mixture was placed at −20°C for 10 min and centrifuged again at 13,000 rpm for 15 min. After discarding the supernatant, the sample was washed twice with 75% ethanol, centrifuged at 13,000 rpm for 5 min at 4°C, and finally dissolved in ddH_2_O.

### 2.9 Transcriptome sequencing and analysis

For the construction of RNA-seq library of testicular tissue, RNA was extracted from testicular tissue, and the concentration of RNA was detected by Qubit (n = 3 each group). Starting with an initial RNA quantity of 500 ng, the KAPA Stranded RNA-Seq Kits (Cat# 07962169001) were utilized to construct the RNA-seq library.

For the 2-cell embryos, the zona pellucida was digested using the PE enzyme, and approximately 10–20 single-cell blastomeres were used to construct each library. The polar body was removed, and the embryo was separated into individual blastomeres using an oral pipette. These blastomeres were washed 2–3 times with 0.5% BSA-PBS. Individual 2-cell blastomeres were then transferred into pre-cooled low-adsorption PCR tubes containing lysis buffer using the oral pipette, followed by gentle mixing and centrifugation. The detailed methodology for RNA sequencing (RNA-seq) library construction is available in previously published articles. For sequencing, the constructed library was run on the NovaSeq 6000 S4 platform using paired-end 150 bp sequencing.

### 2.10 RNA-seq data processing and normalization

Adapters were removed from the RNA-seq data, and low-quality reads were trimmed using Trim_Galore (version 0.6.4) with default parameters. The processed reads were mapped to the mm10 reference genome using hisat2 (version 2.2.1) (Kim et al., 2019). The expression levels of all RefSeq genes were quantified in fragments per kilobase million (FPKM) using StringTie (v1.3.1c) (Shumate et al., 2022). Gene read counts were calculated using FeatureCounts (version 2.0.0) (Liao et al., 2014), and differential expression analysis was performed with Deseq2 (version 1.26.0) (Love et al., 2014). Genes with an adjusted P-value < 0.05 and a fold change >1.5 were considered differentially expressed.

### 2.11 Gene ontology for functional analysis

Functional annotation was performed using the Database for Annotation, Visualization, and Integrated Discovery (DAVID) Bioinformatics Resource, version 6.8 (Huang da et al., 2009).

### 2.12 Reverse transcription and quantitative RT-qPCR analysis

A total of 30–50 2-cell embryos derived from sperm exposed to polystyrene and ddH_2_O was used to obtain RNA by the phenol-chloroform extraction method. cDNA was synthesized using 5X All-in-One RT MasterMix (abm). Quantitative reverse transcription-quantitative polymerase chain reaction (RT-qPCR) was performed using SYBR Premix ExTaq (Takara), and the signals were detected with the ABI 7500 Real-Time PCR System (Applied Biosystems). Glyceraldehyde 3-phosphate dehydrogenase (*Gapdh*) was used as an endogenous control. RT-qPCR were plotted and analyzed using Prism 7.0 software (GraphPad Software, La Jolla, CA, United States).

### 2.13 Statistical analysis

RT-qPCR were plotted and analyzed using Prism 7.0 software (GraphPad Software, La Jolla, CA, United States). Unpaired t-test, ^∗∗∗∗^ *p*-value < 0.0001, ^∗^ *p*-value < 0.05. Details of the other statistical data involved in this paper, including the statistical testing methods used and the number of samples required for the statistics, can be found in the figure legends.

## 3 Results

### 3.1 Transcriptomic analysis of testicular tissue in male mice exposed to polystyrene nanoplsatics

**FIGURE 1:**
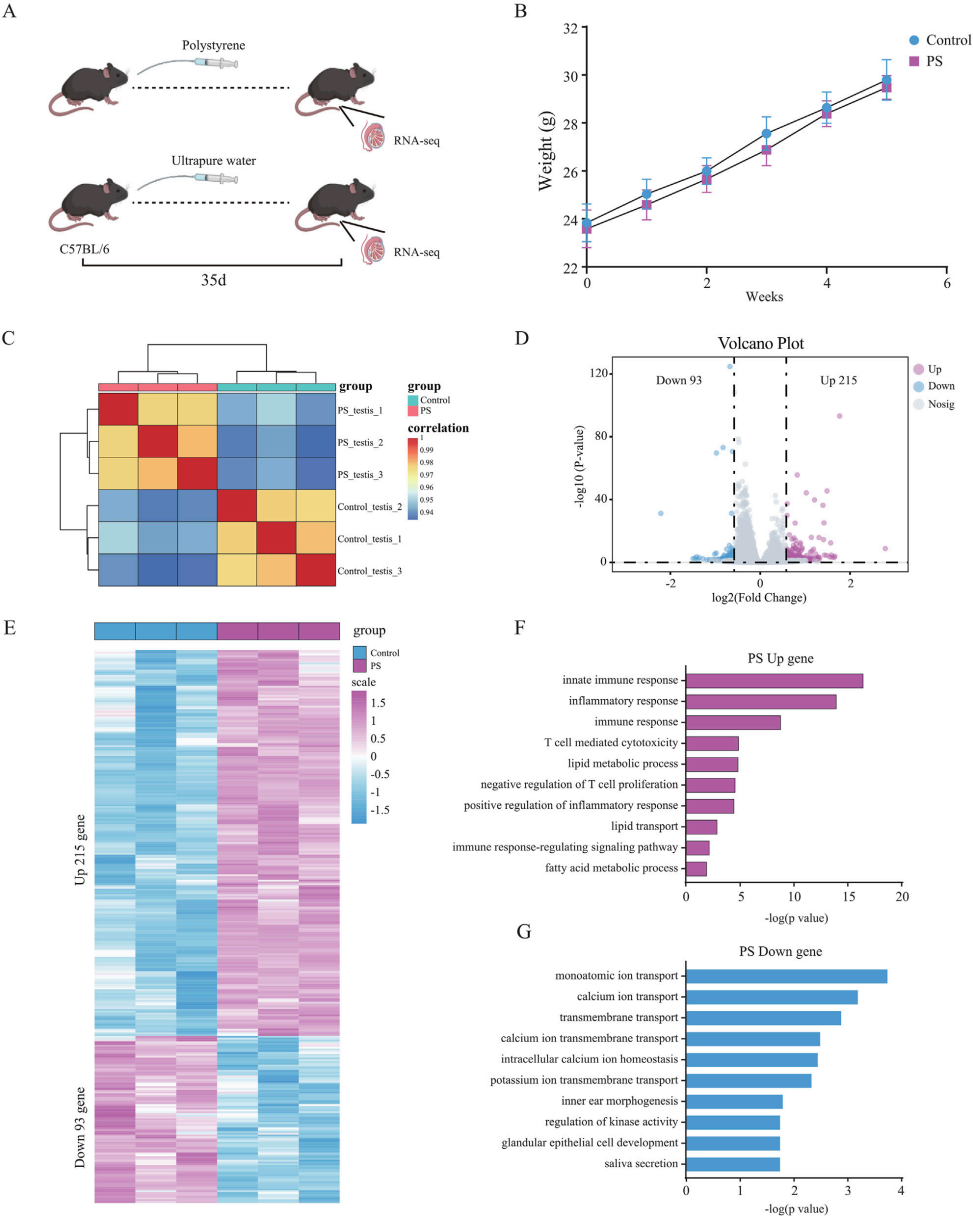
Differential gene expression and functional analysis of testicular tissue in male mice exposed to polystyrene **(A)**. Schematic diagram of male mice exposed to ultrapure water (Control) and polystyrene (PS). **(B)** Body weight statistics of 10- to 12-week-old male C57BL/6 mice oral gavage with ultrapure water or polystyrene (n = 10 each group). **(C)** Correlation analysis of transcriptome data between the Control and PS groups. **(D)** Volcano plot analysis of differentially expressed genes between Control and PS groups. **(E)** Heatmap showing the expression of 308 differential genes in the Control and PS groups. **(F, G)** Gene ontology analysis of upregulated genes (log2 FC > 0.58 and *p*-value < 0.05) **(F)** and downregulated genes (log2 FC < −0.58 and *p*-value < 0.05) **(G)** in the Control and PS groups.

To investigate the effects of polystyrene nanoplsatics on the reproductive system of male mice, 10- to 12-week-old male mice were exposed to polystyrene via oral gavage at a dose of 20 mg/kg/day for 35 days, as illustrated in the schematic diagram (Figure 1A). Weekly body weight measurements were taken for male mice in both the polystyrene (PS) and ultrapure water (Control) groups. Although a slight decrease in body weight was observed in the PS group, this change was not statistically significant when compared to the Control group (Figure 1B). In addition, we conducted data analysis on the weight of testicular tissue and the ratio of testicular weight to body weight after 35 days of treatment, and found that there was no significant difference between the Control and the PS group (Supplementary Figure S1A). However, we found partial atrophy of seminiferous tubules in the testes of the PS group through H&E staining of the transverse section of testicular tissue, indicating that exposed to polystyrene may have an impact on the reproductive health of male mice (Supplementary Figure S1B).

Subsequently, RNA-seq was performed on testicular tissue from both the PS and Control groups. The transcriptome data showed good reproducibility, confirming its suitability for downstream analyses (Figure 1C). A total of 308 differentially expressed genes were identified, with 215 genes upregulated and 93 genes downregulated in the testes of mice exposed to polystyrene (Figures 1D, E; Supplementary Table S1). Gene Ontology (GO) analysis revealed that the upregulated genes were predominantly associated with innate immune response, inflammatory response, T cell-mediated cytotoxicity, lipid metabolic processes, and negative regulation of T cell proliferation. Conversely, the downregulated genes were enriched in processes related to monoatomic ion transport, calcium ion transport, transmembrane transport, and calcium ion transmembrane transport, suggesting disruptions in testicular tissue function due to polystyrene exposure, particularly affecting immune and metabolic pathways (Figures 1F, G).

In summary, our findings indicate that exposure to polystyrene induces gene expression alterations in the testicular tissue of male mice, with significant impacts on biological functions such as immune inflammation and lipid metabolism.

### 3.2 Untargeted metabolomics analysis of male mice sperm exposed to polystyrene

**FIGURE 2:**
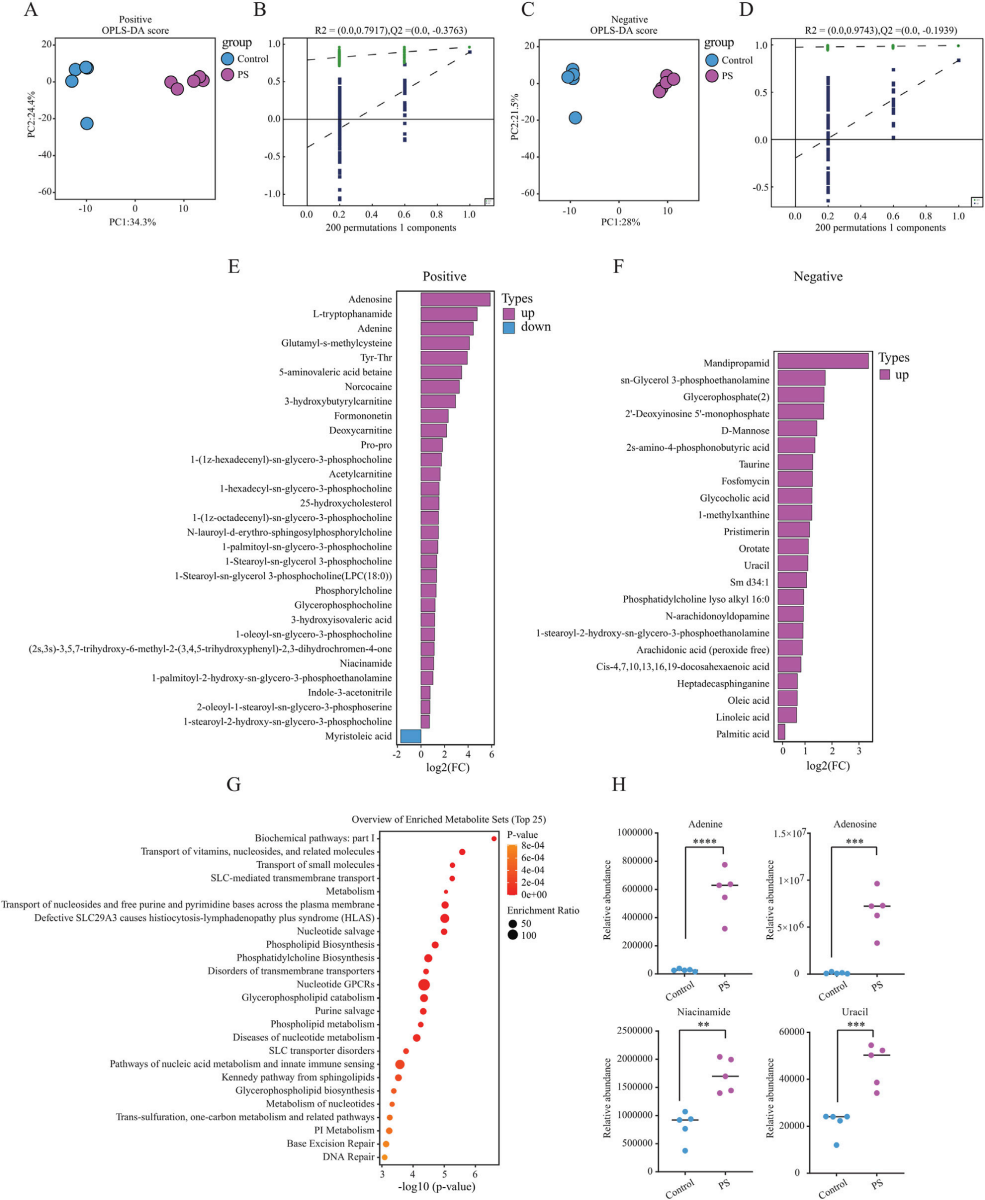
Untargeted metabolomics analysis in male mice between the Control and PS groups **(A, B)**. OPLS-DA model displaying sperm metabolites in male mice exposed to ultrapure water (Control, n = 5) and polystyrene (PS, n = 5) in positive-ion mode **(A)**. OPLS-DA model validation by permutation test **(B)**. **(C, D)** OPLS-DA model displaying sperm metabolites in male mice exposed to ultrapure water (Control, n = 5) and polystyrene (PS, n = 5) in negative-ion mode **(C)**. OPLS-DA model validation by permutation test **(D)**. **(E)** Bar plot showing the differentially expressed metabolites in the positive-ion mode group. **(F)** Bar plot showing the differentially expressed metabolites in the negative-ion mode group. **(G)** Enrichment pathway analysis of KEGG for differential metabolites between the Control and PS groups. **(H)** Levels of differential metabolites in DNA damage-related metabolic pathways observed in both the Control and PS groups (Unpaired t-test, ****P < 0.0001, ***P < 0.001, **P < 0.01).

To investigate the potential effects of polystyrene exposure on sperm metabolism, male mice were exposed to ultrapure water or polystyrene for 35 days (n = 10 per group). Five male mice were randomly selected from each group for untargeted metabolomics analysis of sperm. OPLS-DA analysis revealed significant discrimination between sperm from male mice exposed to ultrapure water and polystyrene, both in positive-ion (Figures 2A, B) and negative-ion (Figures 2C, D) modes. To prevent overfitting of the OPLS-DA model, permutation tests were performed.

In the analysis of differential metabolites in positive-ion mode, 31 significant metabolites were identified, with 30 upregulated and 1 downregulated in the sperm from polystyrene-exposed mice. In the negative-ion mode, 23 differential metabolites were detected, all of which were upregulated in the sperm of mice exposed to polystyrene (Figures 2E, F; Supplementary Table S2). KEGG pathway analysis of the differential metabolites revealed enrichment in metabolic pathways related to the transport of vitamins, nucleosides, and related molecules, glycerophospholipid catabolism, nucleic acid metabolism, innate immune sensing, base excision repair, and DNA repair (Figure 2G). Additionally, metabolites associated with DNA damage, such as adenine, adenosine, niacinamide, and uracil, were identified in both the Control and PS groups (Figure 2H).

In summary, significant differences were observed in the metabolic profiles of sperm exposed to polystyrene. These metabolic alterations in sperm may influence early embryonic development and disrupt gene expression in pre-implantation embryos.

### 3.3 Exposure of male mice to polystyrene leads to decreased sperm motility and early embryonic development after fertilization

**FIGURE 3:**
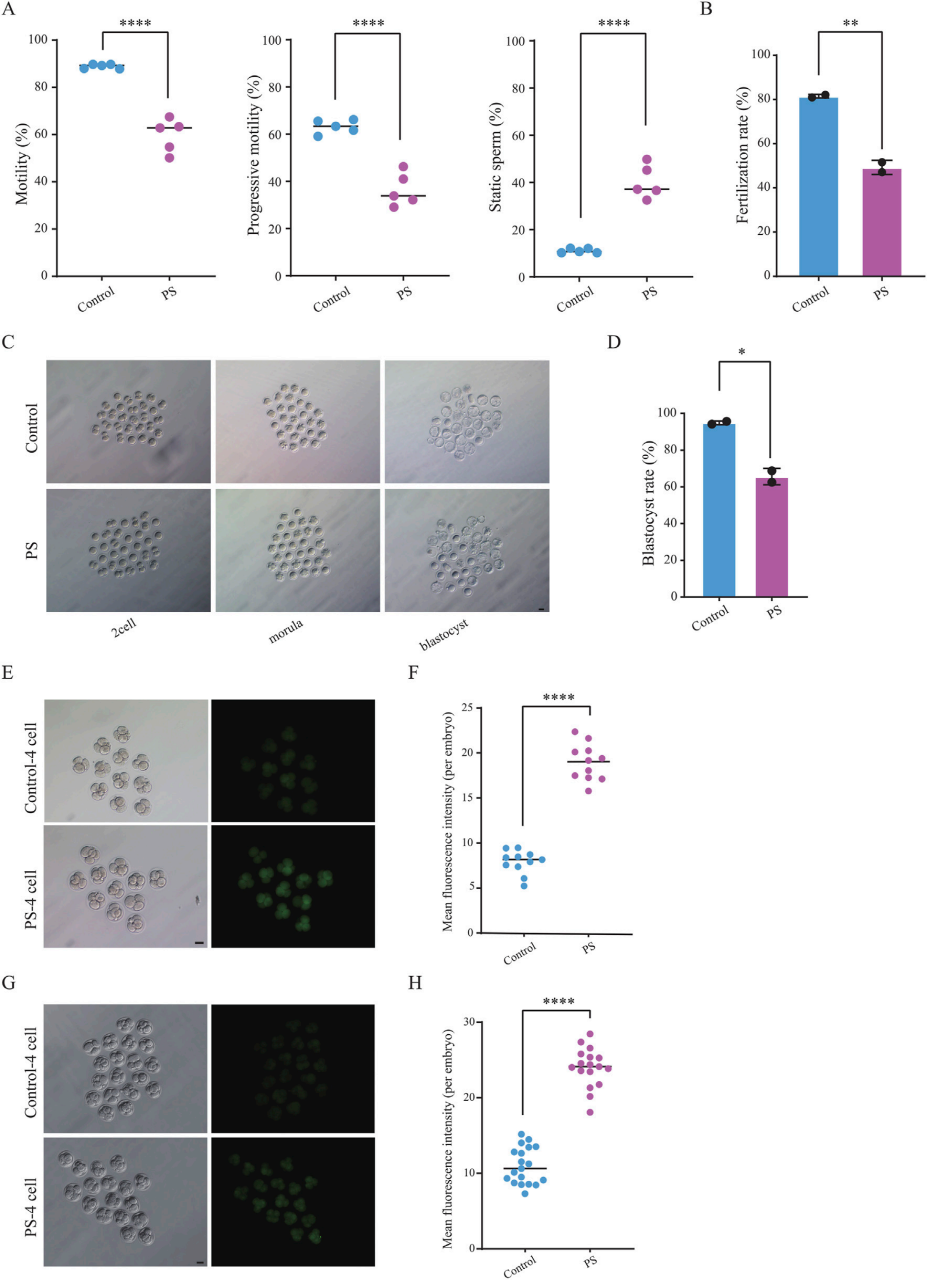
Detection of sperm motility and embryonic reactive oxygen species (ROS) in male mice exposed to polystyrene **(A)**. Total motility, progressive motility, and proportion of static sperm in male mice from the Control and PS groups (Unpaired t-test, ****P < 0.0001). **(B)** Bar plot showing sperm fertilization capacity testing for the Control and PS groups (Unpaired t-test, **P < 0.01). **(C)** Representative images of pre-implantation development in the Control and PS groups (scale bar = 50 µm). **(D)** Bar plot showing the blastocyst ratio in the Control and PS groups (Unpaired t-test, *p-value < 0.05). **(E, F)** Bright-field and ROS fluorescent images of four-cell embryos (n = 11 in each group) **(E)**. ROS fluorescence intensity quantified using ImageJ software **(F)** (scale bar = 50 μm; Unpaired t-test, ****P < 0.0001). **(G-H)**. TUNEL staining during the four-cell stage (n = 19 in Control group and n = 17 in PS group) **(G)**. Fluorescence intensity quantified using ImageJ software **(H)** (scale bar = 50 μm; Unpaired t-test, ****P < 0.0001).

Polystyrene exposure in male mice disrupts testicular gene expression and sperm metabolism, prompting further examination of its impact on sperm quality, including the detection of reactive oxygen species (ROS), motility and fertilization capacity. Computer-assisted sperm analysis (CASA) revealed a significant decrease in sperm motility, aligning with prior studies on the effects of microplastics on male reproduction (Contino et al., 2023; Hu et al., 2024; Zhang et al., 2024). We found that male mice exposed to polystyrene had increased ROS levels in sperm, which may lead to oxidative stress in the sperm (Supplementary Figures S2A, B). Meanwhile, progressive motility of sperm from polystyrene-exposed mice decreased by over 20%, while the proportion of static sperm increased markedly (Figure 3A). Fertilization capacity was assessed through IVF using sperm and oocytes from 8-10-week-old female C57BL/6 mice, revealing a significant reduction in the fertilizing ability of polystyrene-exposed sperm (Figure 3B). Furthermore, when fertilized eggs were cultured to the blastocyst stage, the blastocyst formation rate in the polystyrene-exposed group was also significantly reduced (Figures 3C, D).

Disruption of metabolic pathways related to DNA damage repair was identified in sperm from polystyrene-exposed mice through untargeted metabolomics. To further investigate potential oxidative DNA damage, ROS levels were measured in 4-cell embryos (Figure 3E). The results revealed an increased ROS fluorescence signal intensity in sperm-derived 4-cell embryos exposed to polystyrene (Figure 3F). In addition, we also performed TUNEL staining on 4-cell embryos and found that the fluorescence signal intensity was higher in the exposed group, indicating the presence of significant apoptosis and DNA damage in the embryos (Figures 3G, H).

Overall, polystyrene exposure in male mice impaired sperm motility and fertilization capacity, while also hindering pre-implantation embryo development and elevating oxidative damage. These results suggest that polystyrene exposure in male mice can adversely affect early embryo development via sperm-mediated pathways.

### 3.4 Polystyrene exposure in male mice leads to disruption of early embryonic RNA expression profile

**FIGURE 4:**
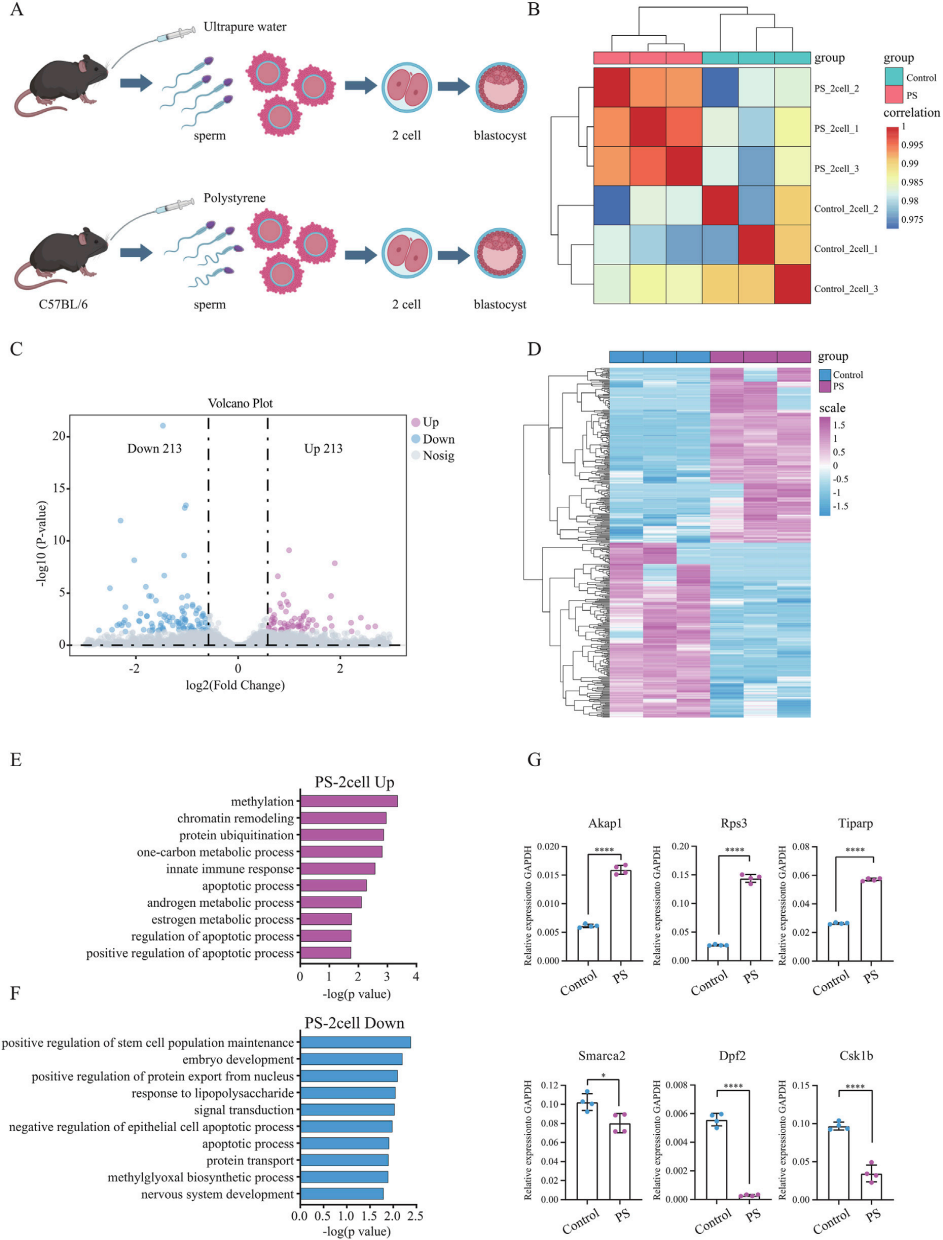
Differential gene expression and functional analysis of sperm-derived 2-cell embryos in male mice exposed to polystyrene **(A)**. Schematic diagram of sperm-derived embryos in male mice exposed to ultrapure water (Control) and polystyrene (PS). **(B)** Correlation analysis of transcriptome data from 2-cell embryos between the Control and PS groups. **(C)** Volcano plot analysis of differentially expressed genes between the Control and PS groups in 2-cell embryos. **(D)** Heatmap showing the expression of 426 differential genes in 2-cell embryos from the Control and PS groups. **(E, F)** Gene ontology analysis of upregulated genes (log2 FC > 0.58 and *p*-value < 0.05) **(E)** and downregulated genes (log2 FC < −0.58 and p-value < 0.05) **(F)** in 2-cell embryos from the Control and PS groups. **(G)** RT-qPCR results for *Akap1*, *Rps3*, *Tiparp*, *Smarca2*, *Dpf2*, and *Csk1b* in 2-cell embryos from the Control and PS groups. (Unpaired t-test, ****p-value < 0.0001; *p-value < 0.05).

Polystyrene exposure in male mice not only disrupted pre-implantation embryo development but also led to an increase in ROS in the embryos, potentially contributing to oxidative stress and DNA damage. To further explore this, Smart-seq2 sequencing was performed on sperm-derived late-stage 2-cell embryos from the polystyrene and Control groups (Figure 4A). Correlation analysis demonstrated high biological replication in the 2-cell RNA-seq data (Figure 4B). A total of 426 differentially expressed genes were identified, with 213 upregulated in the sperm-derived 2-cell transcriptome of polystyrene-exposed male mice (Figures 4C, D; Supplementary Table S3). Gene ontology analysis revealed that the upregulated genes were enriched in processes such as methylation, chromatin remodeling, protein ubiquitination, innate immune response, apoptosis and androgen metabolism. Conversely, downregulated genes were associated with stem cell population maintenance, embryonic development, positive regulation of nuclear protein export, lipopolysaccharide response, and negative regulation of epithelial apoptosis (Figures 4E, F). RT-qPCR further confirmed significant gene expression disturbances in 2-cell embryos derived from sperm exposed to polystyrene. Notably, genes involved in apoptosis (*Akap1* and *Rps3*), androgen metabolism (*Tiparp*), stem cell maintenance (*Smarca2* and *Dpf2*), and embryonic development (*Csk1b*) exhibited altered expression (Figure 4G; Supplementary Table S4).

In summary, the expression of genes in sperm-derived early embryos from male mice exposed to polystyrene was significantly disturbed, which may contribute to reduced pre-implantation embryo development. These findings provide important insights for understanding how polystyrene exposure affects the transition from sperm to embryo.

## 4 Discussion

Recent studies have increasingly highlighted the impact of microplastics, such as polystyrene, on living organisms. Exposure to polystyrene, in particular, has been shown to adversely affect the reproductive health of offspring (Xu K. et al., 2024; Xue et al., 2024). In male mice, polystyrene exposure leads to significant reductions in sperm count and motility, alongside a marked increase in sperm deformity rate (Wen et al., 2023; Xie et al., 2020). Previous research indicates that exposure to microplastics decreases the activity of key testicular metabolic enzymes, including succinate dehydrogenase (SDH) and lactate dehydrogenase (LDH), and reduces serum testosterone levels (Zhu et al., 2014). However, the effects of polystyrene exposure on sperm metabolism remain poorly understood. Notably, significant metabolic disruptions were observed in the sperm of polystyrene-exposed male mice, particularly in pathways related to glycerophospholipid metabolism, nucleic acid metabolism, innate immune sensing and DNA damage repair.

Unlike female mice, the effects of polystyrene exposure on the offspring of male mice remain relatively underexplored (Chen J. et al., 2024; Chen X. et al., 2024; Dou et al., 2024). Polystyrene microplastics disrupt maternal-fetal immune balance and induce reproductive toxicity in pregnant mice (Hu et al., 2021). Exposure to polystyrene microplastics (PS-MPs) during pregnancy can impair endometrial decidualization in the parental generation, alter pregnancy outcomes, and compromise endometrial decidualization in the offspring. Notably, the levels of alkyl-phosphatidylcholine, vinylphosphatidylcholine, and vinylphosphatidylethanolamine in the liver and plasma of male offspring exposed to polystyrene were significantly elevated. Lipid metabolism disruption also displays dose-dependent, gender-specific and tissue-specific patterns (Deng et al., 2022).

Glycerophospholipids possess potent antioxidant properties, capable of neutralizing free radicals, slowing cellular aging and enhancing immune function (Gomes et al., 2016; Mata-Campuzano et al., 2015). In this study, untargeted metabolomics analysis was employed, with a particular focus on the glycerophospholipid metabolism pathway. In the following research, we will conduct targeted metabolism experiments for further investigation. Previous research indicates that both phospholipid synthesis and DNA damage repair are intricately linked to cell cycle progression, however, their potential interactions warrant further investigation (Mingyao et al., 2024; Ovejero et al., 2022).

Exposure to polystyrene significantly impacts early embryonic development and gene expression in male mouse sperm, which is essential for understanding the transgenerational effects of polystyrene. Our findings indicate that polystyrene exposure in male mice elevates ROS levels in embryos, overwhelming the cellular antioxidant system and inducing oxidative stress (Schieber and Chandel, 2014; Xu X. et al., 2024). Additionally, reduced pre-implantation embryo development in male mice exposed to polystyrene suggests biological disruptions, such as DNA damage in the embryos. It remains unclear whether polystyrene exposure also induces epigenetic modifications, including DNA methylation and histone modifications in sperm, which may further alter epigenetic profiles in embryos and disrupt gene expression (Dou et al., 2024; Malinowska et al., 2024).

This study provides an untargeted metabolomics analysis of sperm from male mice exposed to polystyrene, examining its impact on sperm metabolic profiles and investigating its influence on pre-implantation embryo development and gene expression. These findings offer new insights for future research on the effects of polystyrene exposure on offspring. However, this study also has certain limitations, when studying the effects of exposure to polystyrene on male reproductive health, especially the effects of sperm on early embryos, we should also pay attention to the fact that sperm maturation in the epididymis also requires a certain amount of time, rather than just considering the exposure of a 35 days spermatogenic cycle. Next, we will conduct controls at different time periods, such as 20, 40 and 60 days, to study the effects of polystyrene on male reproductive health. In addition, we need more precise research methods to explore how specific genetic or epigenetic changes in sperm caused by exposure to polystyrene affect offspring development.

## Data Availability

The datasets presented in this study can be found in online repositories. The names of the repository/repositories and accession number(s) can be found in the article/Supplementary Material.

## Supplementary material

SUPPLEMENTARY FIGURE S1 Detection of testicular weight and body weight of male mice exposed to polystyrene. **(A)** Detection of testicular weight and the ratio of testis weight to body weight in Control and PS groups of male mice (n = 5 per group). (Unpair t-test, ns represents non-significant). **(B)** H&E staining of the transverse section of testicular tissue in Control and PS groups of male mice (n = 3 per group).

SUPPLEMENTARY FIGURE S2 Intracellular ROS in mouse sperm analysed by fluorescence microscopy using the DCFH-DA probe in different treatment groups. **(A)** Detection of reactive oxygen species (ROS) in sperm of Control group mice (n = 3). **(B)** Detection of reactive oxygen species (ROS) in sperm of PS group mice (n = 3).

SUPPLEMENTARY TABLE S1 Differential gene sets in the testicular tissue of male mice exposed to polystyrene and ultrapure water.

SUPPLEMENTARY TABLE S2 Differential metabolites in the sperm of male mice exposed to polystyrene and ultrapure water.

SUPPLEMENTARY TABLE S3 Differential gene sets in sperm-derived 2-cell embryos exposed to polystyrene and ultrapure water.

SUPPLEMENTARY TABLE S4 Sequences of RT-qPCR primers.
