# Supplementary figures and images for: Exposure to polystyrene nanoplastics impairs sperm metabolism and pre-implantation embryo development in mice
Source: Front Cell Dev Biol. 2025 Feb 28;13:1562331. doi: 10.3389/fcell.2025.1562331 (PMC11906707; doi:10.3389/fcell.2025.1562331)

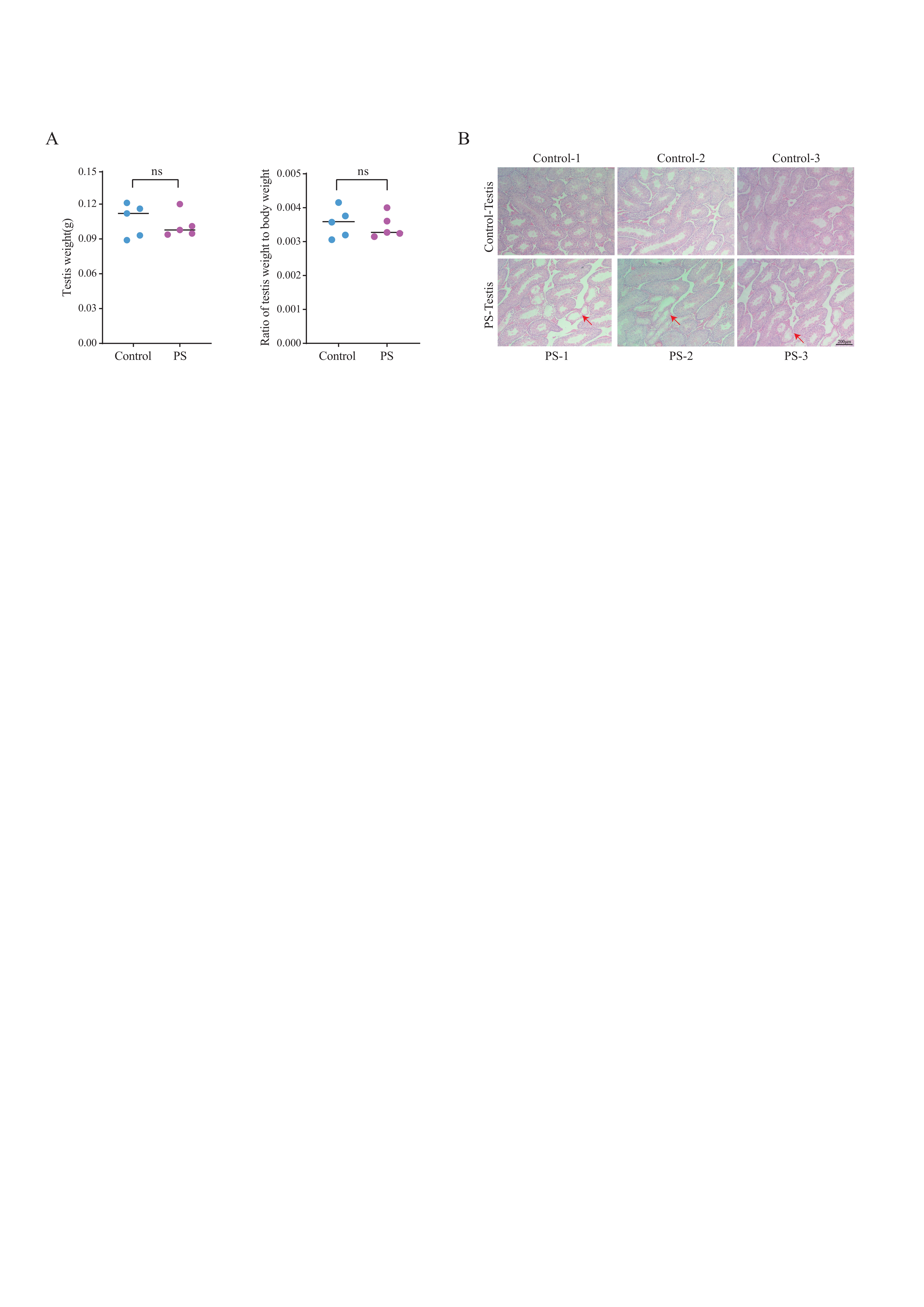

Supplement: Supplementary file 3 [file Image1.jpeg]

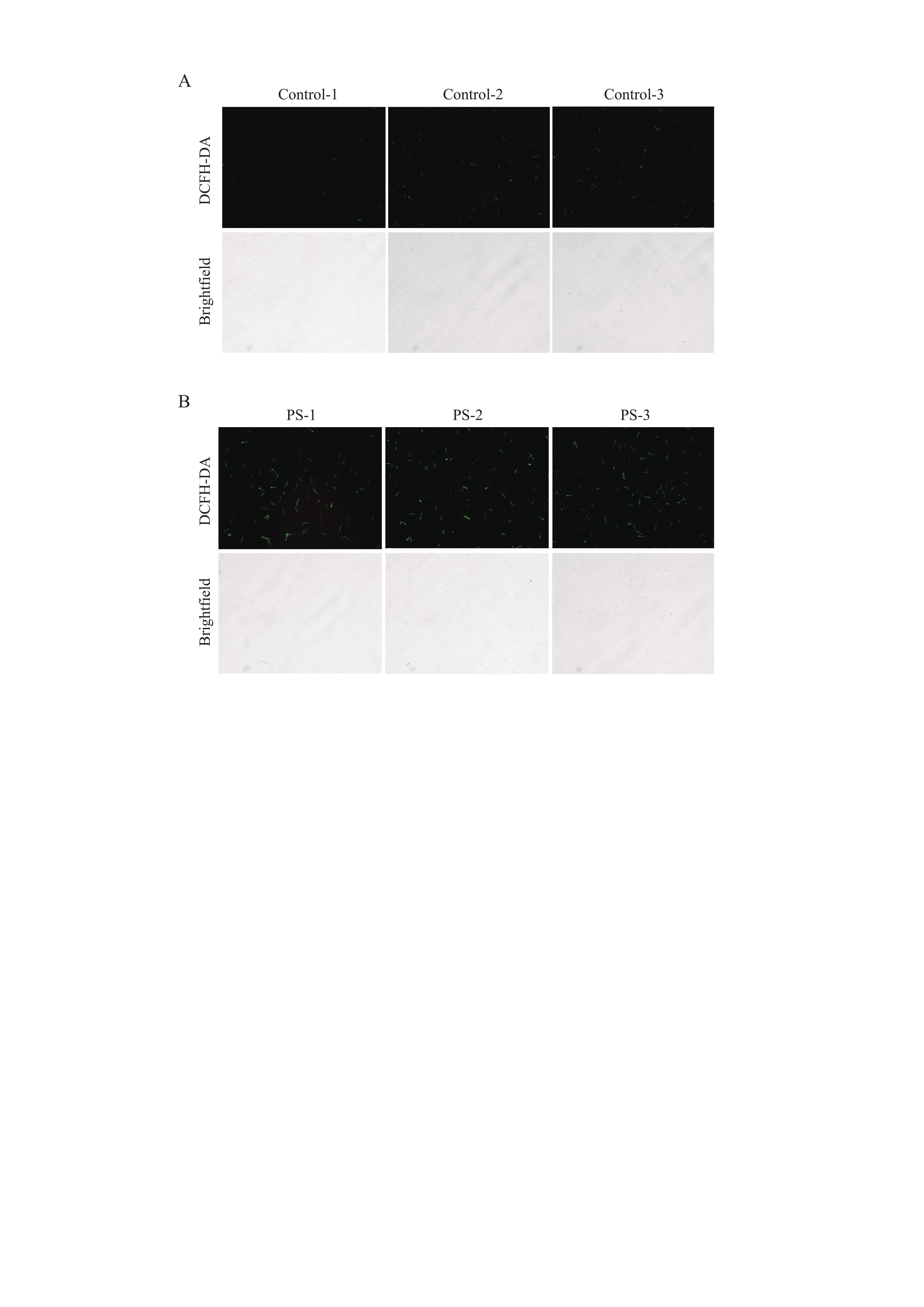

Supplement: Supplementary file 4 [file Image2.jpeg]
